# Healthcare professionals’ views about delivering a rehabilitation programme for individuals living with Atrial Fibrillation: a cross – sectional survey

**DOI:** 10.1186/s13102-024-01000-6

**Published:** 2024-11-05

**Authors:** Munyra Alhotye, Rachael Evans, Andre Ng, Sally J. Singh

**Affiliations:** 1 Present Address: Department of Respiratory Sciences, University of Leicester, Leicester, UK; 2 Department of Respiratory Therapy, King Saud Bin Abdulaziz University for Health Sciences, Riyadh, Kingdom of Saudi Arabia; 3 Department of Cardiovascular Sciences, University of Leicester, Leicester, UK

**Keywords:** Rehabilitation programme, Atrial fibrillation, Healthcare professionals

## Abstract

**Background:**

People living with Atrial Fibrillation (AF) often experience symptoms such as irregular heartbeat, shortness of breath, and fatigue, which can significantly limit their physical activity and overall quality of life. The existing approach to managing AF predominantly revolves around medication and medical procedures, and no prescription of tailored rehabilitation program (RP) is currently offered for this population.

**Aims:**

This study aims to gauge the perspectives of healthcare professionals regarding the implementation of a personalised RP for individuals living with AF and to identify the barriers hindering the referral process.

**Methods:**

A cross-sectional online survey was conducted among healthcare professionals in the UK responsible for caring for adults with AF. The survey consisted of twelve questions designed to uncover healthcare professionals' views on RP for individuals with AF.

**Results:**

A total of 209 respondents participated in the survey, with 57% being female and 43% identifying as specialist arrhythmia nurses. A significant majority (61%) of the participants expressed agreement that an RP could help individuals with AF regain their ability to carry out daily activities, and 58% believed that RP could effectively alleviate symptoms such as breathlessness and palpitations (52%). Virtually all respondents (99%) recommended that a tailored program should encompass education about AF, weight management, and symptom control (94%). Notably, the primary factor influencing their decision to make a referral was the low physical activity levels (80%). Transportation emerged as the chief obstacle to referring patients to the program (62%). A substantial majority (79%) favoured a home-based rehabilitation program as the optimal mean of delivery.

**Conclusions:**

The responses from healthcare professionals reflect a keen interest in implementing a program tailored to individuals with AF, with patients' low physical activity levels being the primary motivator for referrals. Home-based rehabilitation was the preferred mode of delivery, followed by digital interventions.

**Supplementary Information:**

The online version contains supplementary material available at 10.1186/s13102-024-01000-6.

## Background

Atrial fibrillation (AF) is considered the most common sustained type of cardiac arrhythmia [1]. The health and socio- economic burdens related to AF is increasing worldwide [2], and the prevalence of AF is estimated to reach 14 million cases by 2030 in Europe [3]. The incidence of AF is increasing with an aging population [4]. In addition to age, other factors such as obesity, smoking, hypertension, diabetes mellitus, chronic lung diseases, chronic kidney disease, congestive heart failure and valvular heart diseases are associated with development of AF [5, 6]. AF remains the leading cause of stroke, mortality, heart failure, cardiovascular and other thromboembolic events [7–9].

Individuals with AF tend to have frequent symptoms of palpitations, chest pain, shortness of breath, fatigue and dizziness [10, 11]. Moreover, recent evidence has reported that AF related symptoms can cause a reduction in functional capacity and exercise intolerance [12, 13].

The current management and treatment of AF concentrates mainly on antiarrhythmic medication to control heart rate and restore normal sinus rhythm, reducing symptoms, alongside cardiovascular and thromboembolic complications related to the disease [14]. However, AF episodes may not be wholly managed by the administration of this medication to control rhythm disturbance [9]. The clinical guidelines suggested using an invasive procedure called a radiofrequency catheter ablation as an alternative modality to treat and manage AF [8]. Previous systematic review demonstrated that catheter ablation procedure had a positive effect in reducing the recurrence of AF episodes compared to pharmacological therapies. However, the evidence was limited regarding the long term effect of this procedure [15]. Studies highlighted that people living with AF tend to have an impaired quality of life compared to the healthy population, or people with other cardiovascular conditions [16].

In addition, high anxiety levels are usually present in these individuals due to their lack of knowledge and skills in disease self-management about AF related symptoms [17]. Moreover, individuals reported that they have a lack of disease knowledge and have not received education about how to manage and live with AF [18].

Cardiac rehabilitation (CR) is a multidisciplinary comprehensive programme targeting individuals with different cardiovascular diseases. The programme includes clinical assessments, disease education, nutritional counselling, exercise training, behaviour modification and risk factor management [19].The programme is designed to improve the physical and overall well-being of these individuals [20], and it has shown positive effects in improving cardiac symptoms, exercise capacity and quality of life in individuals with AF [21]. However, the evidence about the benefits of the programme for these individuals is limited.

Despite the existing evidence supporting the benefits of rehabilitation programme in individuals with AF, it is not currently offered in any routine care pathways for this population [8, 22, 23]. In addition, the perceptions of healthcare professionals (HCP) toward delivering a tailored rehabilitation programme for people living with AF have not yet been explored. Since referring individuals to rehabilitation programme is usually done by HCPs, it is crucial to gain an insight about their views regarding delivering a comprehensive rehabilitation programme for individuals with AF. Therefore, the aims of this study were to understand the views and opinions of healthcare professionals about delivering a tailored exercise / cardiac rehabilitation programme to people living with AF, and to explore the barriers in referral to this programme.

## Methods

### Study design and settings

This study was a cross-sectional survey conducted through the online platform (Survey Monkey) during the period between July and November 2021 for HCPs who are involved in caring for individuals with AF. We acknowledge that this time frame may have implications for how HCPs perceived participation due to the impact of the second wave of COVID-19 pandemic. The link to the survey was published through professional networks for health professionals working with individuals with cardiac diseases, including BCS (British Cardiovascular Society), and BACPR (British Association for Cardiovascular Prevention and Rehabilitation).

The survey was also published on a social network platform (Twitter) to obtain a greater pool of responses from clinicians working in the United Kingdom (UK). All the data collected from the survey were anonymous with no identifiable information collected.

This survey involved ten multiple-choice questions with additional space for other comments, the survey questions were structured and formulated by the research team based on a previous survey study [24] and validated by experts in the field of cardiac management and rehabilitation based on the current practice and available literature (Supplement 1).

Before the distribution of the survey, the validity of the contents was evaluated and confirmed after piloting the survey among sixteen HCPs with a clinical background in cardiorespiratory management. Their feedback about the contents of the programme and literature about the barriers related to referral were addressed and the structure of the survey was modified to address these changes.

Before respondents gained access to answer the survey questions, the overview and aims of the study were stated along with information about the research team.

Completion of the survey questions took approximately 5 to 7 min. The survey tool included two pages with multiple-choice answers in three parts. The structure of the survey included the following parts: Part 1 was asking about the participants' demographic data which includes their gender, professional background, years of clinical experience, responsibilities in the management of individuals with cardiac diseases and if HCPs had previously referred individuals with AF to a rehabilitation programme. Part 2 involved four main questions about HCPs views toward CR. The initial question asked about the best method to deliver CR for individuals with AF, followed by a second question about individuals’ preference for receiving the programme, and participants could choose more than one option. The third question involved four statements regarding their views toward the effectiveness of rehabilitation programme for AF using a 5-point Likert scale tool which ranges from (1 point which indicates strongly disagree to 5 points which indicate strongly agree) and the fourth question was about their opinion toward additional components of rehabilitation programme for AF other than exercise training.

Part 3 involved two main questions regarding factors that influence the decision to refer individuals with AF to rehabilitation programme and the factors that may not influence or prevent HCPs from referring these individuals to the programme **"**no influence, **"** some influence, **"** and **"** strong influence **"** were used as a grading tool.

### Study participants and sampling procedure

The study participants were recruited through a convenience sampling technique. Cardiac physicians, general practitioners, electrophysiologists, cardiac and arrhythmia nurses, physiotherapists, exercise specialists and other HCPs who actively engaged in managing individuals with AF or who had a relevant experience with this population were the main target of our recruitment.

A formal sample size calculation was not required, as this was an exploratory survey aimed to gain a baseline knowledge, initial thoughts, and opinions toward a rehabilitation programme for individuals living with AF, as well as potential barriers in implementing and delivering the programme.

### Statistical analysis

The survey data were collected, uploaded into a spreadsheet and then analysed using the Statistical Package for Social Sciences (SPSS software, Version 25). The characteristics of the study participants and the categorical variables were analysed using descriptive statistics such as frequency (%), mean, and standard deviation (SD).

## Results

Overall, 209 HCPs (119 female (57.0%) responded to the online survey across the U.K between July 22, 2021, and November 3, 2021. The majority of HCPs were specialist arrhythmia nurses (20.5%) and cardiac nurses (19.6%), followed by cardiologists (18.9%) and general practitioners (13.9%) (Table 1). Out of 209, 108 (51.6%) had more than ten years of clinical experience managing those with atrial fibrillation. Ongoing Management 154 (73.7%), non-urgent care 147 (70.3%), and outpatient clinics 134 (64.1%) were the most common responsibilities of HCPs caring for individuals with AF (Table 1).

**Table 1:** Demographic data and Characteristics of all study respondents (*n* = 209)

Demographic variables	Frequency (%)
**Gender**	
Male	90 (43.0%)
Female	119 (57.0%)
**Profession**	
Specialist Arrhythmia Nurse	43 (20.5%)
Cardiac Nurse	41 (19.6%)
Cardiologist	38 (18.9%)
General Practitioner	29 (13.9%)
Physiotherapist	22 (10.5%)
Specialist Respiratory Nurse	18 (8.6%)
Primary Care Nurse	0 (0.0%)
Other	18 (8.6%)
**Years of experience**	
< 1 year	1 (0.5%)
1–2 years	1 (0.5%)
3–4 years	16 (7.6%)
5–6 years	33 (15.8%)
7–8 years	27 (12.9%)
9–10 years	23 (11.0%)
> 10 years	108 (51.6%)
**Responsibilities of caring for individuals with AF**	
Ongoing management	154 (73.7%)
Non-urgent care	147 (70.3%)
Outpatient clinics	134 (64.1%)
Urgent assessments	92 (44.0%)
In patient treatment	90 (43.1%)
Prescribing	80 (38.9%)
Rehabilitation	78 (37.3%)
Admission prevention	77 (36.8%)
Medication check	76 (36.5%)
Oxygen therapy	73 (34.9%)
Diagnosis	72 (34.4%)
Primary care	45 (21.5%)
Other	7 (3.3%)

### Perception on referring individuals with AF to a rehabilitation programme

Out of 209 HCPs, 93 (44.5%) strongly agreed, and 122 (53.6%) agreed that a rehabilitation programme would improve physical fitness for individuals with AF (Table 2). Moreover, 60 (28.7%) strongly agreed, and 123 (58.8%) agreed that a rehabilitation programme would reduce breathlessness. Thirty-seven respondents (17.7%) strongly agreed that a rehabilitation programme would reduce palpitations and fatigue (Table 2). Lastly, 127 (60.7%) strongly agreed, and 80 (38.3%) agreed that a rehabilitation programme would improve individuals’ ability to perform daily activities (Table 2).

**Table 2:** Healthcare professionals’ perception on referring individuals with atrial fibrillation to rehabilitation programme (*n* = 209)

Item	Frequency (%)
**Perception on referring individuals with AF to rehabilitation programme**	
*I think that rehabilitation programme will improve individuals’ physical fitness*	
Strongly agree	93 (44.5%)
Agree	112 (53.6%)
Not sure	3 (1.4%)
Disagree	0 (0%)
Strongly disagree	1 (0.5%)
*I believe that rehabilitation programme would be beneficial in reducing breathlessness*	
Strongly agree	60 (28.7%)
Agree	123 (58.8%)
Not sure	25 (11.9%)
Disagree	1 (0.5%)
Strongly disagree	1 (0.5%)
*I think the programme would benefit in reducing AF-related symptoms including fatigue and palpitation*	
Strongly agree	37 (17.7%)
Agree	108 (51.6%)
Not sure	62 (29.6%)
Disagree	1 (0.5%)
Strongly disagree	1 (0.5%)
*I think the programme would benefit individuals’ in performing their daily activities*	
Strongly agree	127 (60.7%)
Agree	80 (38.3%)
Not sure	1 (0.5%)
Disagree	0 (0.0%)
Strongly disagree	1 (0.5%)

Data are presented as frequencies and percentages

### Referral, mode of programme delivery, and components of the programme

Only 90 (43.1%) HCPs out of 209 had referred individuals with AF to a rehabilitation programme. A total of (57.0%) HCPs had not referred or were not sure if they had ever referred individuals with AF to a rehabilitation programme (Table 3). The preferred methods of delivering rehabilitation programme from HCPs perspective were at-home (79.4%), followed by using virtual online classes (64.6%) and digital programmes (61.7%) (Table 3).

**Table 3:** HCPs perspective on referring individuals with atrial fibrillation to a rehabilitation programme (*n* = 209)

Item	Frequency (%)
**Referral to rehabilitation programme for individuals with AF**	
Yes	90 (43.1%)
No	73 (34.9%)
Not sure	46 (22.0%)
**The best method to deliver the programme for individuals with AF**	
At home	166 (79.4%)
Supervised virtual online classes	135 (64.6%)
Using non supervised digital programme	129 (61.7%)
At the hospital	113 (54.1%)
At a community centre	52 (24.9%)
At home with telephone support	32 (15.3%)
**Thoughts about individuals with AF preference**	
At home	144 (68.9%)
At the hospital	140 (66.9%)
Supervised virtual online classes	78 (37.3%)
Using non supervised digital programme	72 (34.4%)
At a community centre	70 (33.5%)
At home with telephone support	31 (14.8%)
**Components of rehabilitation programme other than exercise**	
Information about atrial fibrillation	206 (98.5%)
Weight management	201 (96.2%)
Symptoms management	197 (94.3%)
Information about medications	170 (81.3%)
Mood management	152 (72.7%)
Smoking cessation	145 (69.3%)
Other	28 (13.4%)

Data are presented as frequencies and percentages

However, HCPs believed that individual’s preference of delivering a rehabilitation programme would be at-home (68.9%), followed by receiving the programme in the hospital (64.6%) and via digital programme (61.7%) (Table 3). Information about atrial fibrillation, weight management and symptoms management were considered the essential components of a rehabilitation programme aside from the exercise component by 206 (98.5%), 201 (96.2%), and 197 (94.3%) HCPs, respectively (Table 3).

### Factors that influence the decision for rehabilitation programme referral

**Fig. 1:**
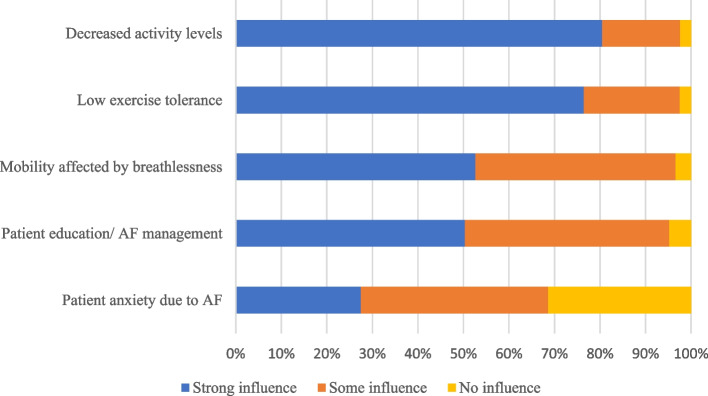
Factors influencing the decision to refer individuals with AF to a rehabilitation programme

The most common associated factors that strongly influenced the decisions of HCPs to refer individuals with AF to a rehabilitation programme were decreased activity levels (80.2%), followed by low exercise tolerance (76.2%), mobility affected by breathlessness (52.6%), and individuals’ education and disease management (50.5%); ( Fig. 1).

### Factors that influence the decision for not to refer to rehabilitation programme

**Fig. 2:**
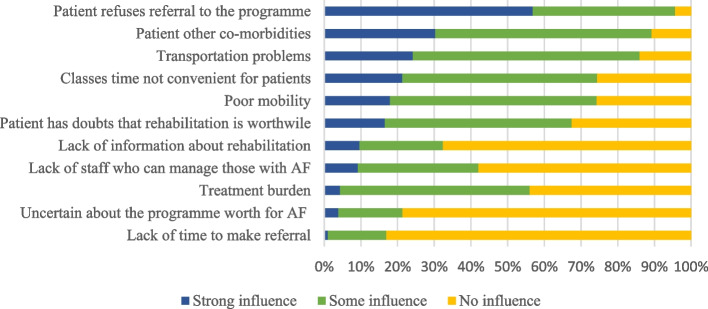
Barriers in referring individuals with AF to a rehabilitation programme

The most common barriers that strongly influenced HCPs decisions not to refer individuals with AF to a rehabilitation programme were patients’ refusing referral (56.8%), followed by co-morbidities (30.2%), transportation problems (24.1%), and Timing of classes not convenient for patient (21.2%); (Fig. 2).

## Discussion

To the best of our knowledge, this is the first study to understand the views of HCPs from different professional background toward the benefits of delivering a comprehensive CR programme and experience in referring or considering a referral to CR programme for individuals with AF in UK. In this nationwide study, HCPs had an overall agreement upon the benefits of CR regardless of their professional background. However, the overall referral rates of individuals with AF to CR programmes were low, which demonstrate a clear lack in the current referral practice. This was mainly attributed to factors related to individuals with AF as the majority refusing the referral to CR programmes and the presence of other co-morbidities which effect the referral process. In addition, home-based rehabilitation programme was considered as the most suitable way to deliver the programme. Information about AF, weight and symptoms management was the most essential components of CR recommended aside from exercise training.

It is widely acknowledged that CR is considered as an effective non-pharmacological intervention for managing individuals with different cardiovascular diseases, including heart failure and coronary artery disease [25, 26]. Participation into these programmes has been shown to be associated with a significant reduction in diseases related symptoms, improves functional and exercise capacity, reduces further cardiac events, hospital admission and enhance the overall quality of life in this population [27–29]. However, the evidence about these benefits for individuals with AF are limited [21]. Although this survey study demonstrated a consensus about the promising benefits of CR for individuals with AF, the referral rates were considered low among HCPs. While this might be expected since the current clinical guidelines for managing AF didn’t include referral to CR [14]. A previous observational study conducted an audit of 145 records of individuals admitted with AF found that only 25% of the total admission was referred to outpatient rehabilitation programme [30], in line with the findings of our study.

Our study showed that individuals with AF were less likely to be referred to rehabilitation programmes, despite the evidence demonstrated its benefit in reducing disease related symptoms and improving functional capacity [31] hence, there need to be a structured multidisciplinary plan to enhance the referral process for those with AF.

It is worthwhile to address that HCPs identified that home-based setting would be the preferred method to deliver a rehabilitation programme for individuals with AF, this might be due to the fact that there is a low uptake of hospital-based programmes in individuals with cardiovascular conditions (42%—44%) across the country [32], highlighted by complex factors related to the organisation and the system of delivering the service [33]. Other factors related to not attending a conventional rehabilitation programme have been identified such as lack of time, difficulties with transportation, work commitments and distance to travel to rehabilitation centres [27, 34, 35]. Importantly, the existence of COVID-19 pandemic may affect the decision of HCPs as most face-to-face rehabilitation services were paused or limited due to social distance measures. In current practice, home based rehabilitation has been adopted as an alternative option to deliver the service for people living with different heart conditions [36] and reports highlighted that individuals with cardiovascular conditions who have employment commitments would prefer using this option [37]. Interestingly, self-delivered programme has shown a positive outcomes equivalent to hospital-based programme [38].

Additionally, there is a considerable interest among HCPs in using digital technology to deliver the service for individuals with AF. The use of digital programmes could permit flexibility of programme delivery, as individuals will be able to complete the programme at a time and place appropriate to them. Recent evidence reported that using web-based can improve health-related quality of life, disease related symptoms, levels of anxiety and depression, exercise capacity in people with cardiovascular conditions [36, 39, 40], which suggest the potential value of this intervention for those with AF.

In this survey, providing information about atrial fibrillation was perceived by HCPs as the most essential component of a comprehensive rehabilitation programme for AF aside from exercise training. This is in line with previous studies stated that educating individuals with AF about arrhythmia, risk factors, current treatments, and attitudes of self-management is considered as a key factor in AF management [41]. When individuals gain a good understanding about their condition they would perceive a good control over AF, report fewer symptoms,, and attribute less anxiety level toward AF [42]. Thus, providing these individuals with information about AF and how to manage it is essential to promote positive outcomes [43]. Therefore, adding an educational component to the proposed structured rehabilitation programme for AF could optimise the benefit from this clinical service.

Decreased activity levels, low exercise tolerance and mobility affected by the nature of AF were the main factors that influence HCPs decision to refer individuals with AF to a rehabilitation programme. It has been reported in the literature that these factors are usually present in those with AF and might lead to impaired quality of daily living and worsen the disease prognosis [44]. A previous Cochrane review demonstrated that exercise-based rehabilitation programme targeted individuals with AF has significantly increased their functional and exercise capacity (standard mean difference (SMD): 0.86, 95% CI 0.46 to 1.26; *n* = 359). However, the study included only six randomized trials and the quality of the evidence was moderate to low. Moreover, these interventions weren’t comprehensive rehabilitation programme [9]. Therefore, there is a need for more trials to inform the clinical benefits of comprehensive rehabilitation programme targeting those with AF.

One of the most commonly selected barriers to rehabilitation delivery in this survey was patient-related factor that individuals with AF refuse to enrol into a rehabilitation programme. This might be due to the lack of knowledge about the benefits of enrolling into these programmes among this clinical population. Moreover, it is mentioned in the literature that individuals with AF are reluctant to do exercise due to the fear of developing exercise-induced episodes of AF such as palpitation, fatigue and shortness of breath [45]. Therefore, its essential to educate individuals about the possible benefits of CR rehabilitation as a way of managing their symptoms and improving their quality of daily living. Therefore, there is a need to conduct further studies to investigate the possible factors affecting the enrolment in this population.

A further barrier to rehabilitation programme delivery for AF reported in this study is patient related comorbidities. Individuals with AF tend to suffer from comorbidities such as hypertension, diabetes, angina and heart failure [46], which could affect participation into the programme. Similar results were found in a previous study investigating the barriers to cardiac rehabilitation programme including those with arrhythmia. Participants with AF reported that their comorbidities could prevent them from enrolling in such a programme [47].

Moreover, difficulties with transportation have been highlighted as a factor affecting the referral process. Similar factor was pointed out by individuals with cardiac disease that distance and travel time to rehabilitation centre were obstacles for attending the programme [35]. Therefore, an alternative and flexible ways to deliver the programme for individuals with AF need to be considered and home-based rehabilitation programmes were favoured by HCPs; this is interesting that is preferred mode as there is very limited evidence for unsupervised rehabilitation in this population.

### Study Limitations

This survey study was exploratory with a convenience sample of HCPs recruited through online methods only, selection bias related to internet access and social network usage are therefore likely to be present. This study was conducted during the second wave of COVID-19 pandemic, which might influence the number of participants and HCPs opinions towards the appropriate mode or rehabilitation delivery. Notably, the absence of input from individuals with AF may represents a gap in understanding their preferences and needs from rehabilitation programme. Moreover, the study would have benefited from an in-depth qualitative study to gain an insight to inform the development of a suitable rehabilitation programme for individuals with AF based on the present findings of this study. However, this survey study was designed to obtain initial opinions, baseline knowledge and thoughts regarding rehabilitation programme for this population.

## Conclusion

The benefits of comprehensive rehabilitation programme for individuals with AF are well recognised among HCPs regardless of their profession. Home-based rehabilitation was the most preferred way to deliver the programme followed by using digital interventions, while education, information about AF and weight management being the most essential components of this programme aside from exercise training. Barriers influenced HCPs in enrolling patients into rehabilitation mainly related to patients’ refusal of referral and presence of comorbidities. However, poor activity levels and exercise tolerance are the main factors to influence the decision to refer individuals with AF to this service. The findings of this survey study will contribute to the implementation and development of a comprehensive rehabilitation programme targeting individuals with this condition and support, enhanced and guide for referral.

## Supplementary Information

Supplementary Material 1.

## Data Availability

All data generated and/or analysed during this study are available from the corresponding author on reasonable request.
