# Supplementary material for: Healthcare professionals’ views about delivering a rehabilitation programme for individuals living with Atrial Fibrillation: a cross – sectional survey
Source: BMC Sports Sci Med Rehabil. 2024 Nov 5;16:227. doi: 10.1186/s13102-024-01000-6 (PMC11536881; doi:10.1186/s13102-024-01000-6)
Supplement: Supplementary file 1 — Supplementary Material 1. [file 13102_2024_1000_MOESM1_ESM.pdf]

Attitudes and Expectations of Health Care Professionals Toward Rehabilitation Programme for Patients with Atrial Fibrillation

Rehabilitation programme is a beneficial intervention to support patients with cardiac diseases. Recent studies found that it could improve exercise capacity and quality of life and reduces disease burden in patients with Atrial Fibrillation(AF).

We are aiming to understand your attitude and expectations toward delivering a rehabilitation programme for this population, and it would be great if you could answer this questionnaire.

I. Demographic Information

1. Gender

❑ M ❑ F ❑ Prefer not to say

2. Your background

❑ Specialist arrhythmia nurse	❑ Primary care doctor
❑ Primary care nurse	❑ Specialist respiratory nurse
❑ GP	❑ Physiotherapist
❑ Cardiac doctor	❑ Other:

3. What responsibilities do you have for the care of people with cardiac diseases? Tick all that apply.

❑ Diagnosis	❑ Prescribing	❑ Inpatient treatment
❑ Non-urgent care	❑ Ongoing management	❑ Outpatient clinics
❑ Urgent assessments	❑ Admission prevention	❑ Primary care
❑ Oxygen therapy	❑ Medication checks	❑ Other

If other, please give details

4. How many years' experience do you have of caring for people with cardiac problems?

III. Perceptions to rehabilitation programme

8. Do you routinely refer patients with AF to cardiac rehabilitation programme?

- ❑ Yes
- ❑ No
- ❑ Not sure

9. Do you think that rehabilitation programme would

	Yes	No	Not sure
- Improve health related quality of life.	❑	❑	❑
- Imrove exercise capacity.	❑	❑	❑
- Reduce AF related symptoms	❑	❑	❑

10. What do you think the best way to deliver a rehabilitation programme for this population. (Tick all that apply).

- ❑ at the Hospital. Where they can follow a programme with the support of health care professionals.
- ❑ at a Community Centre. Where they can follow a programme with the support of community health workers.
- ❑ at Home. Where they can follow a programme manual.
- ❑ by using a digital programme with support from a health care professional to answer thier questions.
- ❑ by participating in a virtual online classes with support from a health care professionals.
- ❑ by following a programme with the support of health care professionals through the phone.

11. What do you think patients would prefer.(Tick all that apply).

- ❑ at the Hospital. Where they can follow a programme with the support of health care professionals.
- ❑ at a Community Centre. Where they can follow a programme with the support of community health workers.
- ❑ at Home. Where they can follow a programme manual.
- ❑ by using a digital programme with support from a health care professional to answer thier questions.
- ❑ by participating in a virtual online classes with support from a health care professionals.
- ❑ by following a programme with the support of health care professionals through the phone.

12. For each statement please select the answer that best suits your opinion.

	Strongly disagree	Disagree	Not sure	Agree	Strongly agree
I think that rehabilitation programme will improve patients Physical Fitness .	❑	❑	❑	❑	❑
I believe that rehabilitation programme would be beneficial in reducing breathlessness.	❑	❑	❑	❑	❑
I think that rehabilitation programme would be beneficial on reducing other symptoms (palpitation, fatigue).	❑	❑	❑	❑	❑
I think that rehabilitation programme would be beneficial on patients ability to perform daily activities.	❑	❑	❑	❑	❑

13. What do you think that rehabilitation programme for AF should contain a side from an exercise?

- ❑ Lifestyle modification
- ❑ Weight managment
- ❑ Stress management
- ❑ Information about disease
- ❑ Symptoms management
- ❑ Smoking cessation
- ❑ Others

If other, please give details

V. Referral to rehabilitation programme

14. What factors might influence your decision to refer a patient to a rehabilitation programme?

	No influence	Some influence	Strong influence
Mobility is affected by breathlessness	❑	❑	❑
Decreasing activity levels	❑	❑	❑
Low exercise tolerance	❑	❑	❑
Patient anxiety related to disease	❑	❑	❑
Patient education and disease management	❑	❑	❑
Fatigue	❑	❑	❑

Others	❑

If other, please give details

15. What factors might influence your decision <u>Not</u> to refer a patient to a rehabilitation programme ?

	No influence	Some influence	Strong influence
I don't have enough information about rehabilitation programme	❑	❑	❑
I'm uncertain that the programme is worthwhile	❑	❑	❑
Patient refuses referral	❑	❑	❑
Patient co-morbidities	❑	❑	❑
Poor mobility	❑	❑	❑
Patient has doubts that rehabilitation is worthwile	❑	❑	❑
Transportation problems	❑	❑	❑
Timing of classes not convenient for patient	❑	❑	❑
Lack of time to make referral	❑	❑	❑
Lack of experienced staff who can manage patients with AF	❑	❑	❑
Other	❑		

If other, please give details

Thank you for your help in answering this material.
